# Time Domain Near Infrared Spectroscopy Device for Monitoring Muscle Oxidative Metabolism: Custom Probe and In Vivo Applications

**DOI:** 10.3390/s18010264

**Published:** 2018-01-17

**Authors:** Rebecca Re, Ileana Pirovano, Davide Contini, Lorenzo Spinelli, Alessandro Torricelli

**Affiliations:** 1 Dipartimento di Fisica, Politecnico di Milano, Piazza Leonardo da Vinci, 32, 20133 Milan, Italy; ileana.pirovano@polimi.it (I.P.); davide.contini@polimi.it (D.C.); alessandro.torricelli@polimi.it (A.T.); 2 Istituto di Fotonica e Nanotecnologie, Consiglio Nazionale delle Ricerche, Piazza Leonardo da Vinci, 32, 20133 Milan, Italy; lorenzo.spinelli@polimi.it

**Keywords:** time domain near infrared spectroscopy, muscle oxidative metabolism, total hemoglobin, tissue oxygen saturation

## Abstract

Measurement of muscle oxidative metabolism is of interest for monitoring the training status in athletes and the rehabilitation process in patients. Time domain near infrared spectroscopy (TD NIRS) is an optical technique that allows the non-invasive measurement of the hemodynamic parameters in muscular tissue: concentrations of oxy- and deoxy-hemoglobin, total hemoglobin content, and tissue oxygen saturation. In this paper, we present a novel TD NIRS medical device for muscle oxidative metabolism. A custom-printed 3D probe, able to host optical elements for signal acquisition from muscle, was develop for TD NIRS in vivo measurements. The system was widely characterized on solid phantoms and during in vivo protocols on healthy subjects. In particular, we tested the in vivo repeatability of the measurements to quantify the error that we can have by repositioning the probe. Furthermore, we considered a series of acquisitions on different muscles that were not yet previously performed with this custom probe: a venous-arterial cuff occlusion of the arm muscle, a cycling exercise, and an isometric contraction of the vastus lateralis.

## 1. Introduction

The development of techniques able to evaluate the role of skeletal muscle mitochondria dysfunction, both in the progression of pathologies and in monitoring of muscle performances, is of relevant interest in particular in vivo evaluations [1,2]. A recent review from Willingham et al. [3] highlights recent studies related to the in vivo assessment of skeletal muscle mitochondrial dysfunction in clinical human populations and addresses the potential for clinical translation of the findings, in particular employing a non-invasive optical technique: the near-infrared spectroscopy (NIRS).

NIRS [4] is a spectroscopic technique that makes use of light (660–1100 nm) to non-invasively monitor tissue hemodynamics and oxidative metabolism. Studying the attenuation of light inside the tissue and exploiting the differences in the absorption spectra of the oxy- (O_2_Hb) and deoxy- (HHb) hemoglobin, it is possible to monitor the changes of this two specimen and then to calculate the total hemoglobin content (tHb = HHb + O_2_Hb) related with blood volume and tissue oxygen saturation (SO_2_ = O_2_Hb/tHb).

The oxygen consumption can be measured with NIRS during ischemia periods [5], to evaluate exercise-induced adaptations in muscle mitochondrial function [6], to measure muscle mitochondrial function in clinical populations that may be affected by declines in oxidative capacity, and to evaluate the effectiveness of therapeutic interventions targeting muscle mitochondrial function [7]. Again, in other review papers, the NIRS potentiality is underlined, from an exercise physiology point of view, to reflect the fractional oxygen extraction in the investigated tissue volume for applications on athletes, healthy subjects, and patients during bed-rest period or rehabilitation steps [8,9,10,11,12]. Moreover, the NIRS technique is often used to monitor muscle oxidative metabolism during short (5–10 s) periods of cuff inflation to block blood flow to the muscle and the following recovery periods. This short ischemia allows to separate muscle oxygen consumption from oxygen delivery, which confounded earlier attempts to use NIRS during the recovery phase [13]. This could be necessary for the Continuous Wave (CW) NIRS approach, which is the most widespread in commercial and laboratory environments. CW NIRS employs steady-state light source (e.g., lamp or LED), which emits constant power in time, and the device measures the total attenuation, which depends on the absorption (μ_a_) and reduced scattering (μ’_s_) coefficients of the examined tissue [14,15]. This approach allows us to build a portable and almost cheap device, but has some technical critical points that at the current stage are not yet solved. The first one concerns with the impossibility to extrapolate absolute values for the O_2_Hb and HHb with just a two-wavelength, one-channel measure. The second one relates with the fact that the acquired signal is referred to the whole tissue investigated under the probe. For muscle measurements, it refers to the skin, the fat layer, the capillary bed, and the muscular tissue. It is not straightforward to separate the contributions of these layers, and the signal is strongly contaminated from superficial physiological effects and moving artifacts.

A different approach, namely the Time Domain (TD) NIRS, could be used to partially overcome these problems. In TD NIRS [16,17], laser light pulses, with a duration of tens or hundreds of picoseconds, are injected into the tissue, and from the modifications in attenuation, shape and delay of laser pulses observed in recollected light, it is possible to extrapolate absolute values for tissue optical properties and, consequently, also for O_2_Hb and HHb concentrations. Furthermore, it is possible to discriminate between contributions to the detected signal coming from different depths. An experimental evidence of the capability of TD NIRS to separate between the contribution coming from the superficial capillary bed and the one from the deeper tissues can be find in our previous work published in 2012 [18]. As a matter of fact, in the review from Grassi et al. [12], a comprehensive list of the commercial muscle oximeters present on the market in 2016 is presented, and only one is based on TD NIRS: the tNIRS-1 from Hamamatsu, Japan, which to our knowledge is currently not sold outside Japan and has replaced the old TRS-20 device. The Authors have previously successfully used TD NIRS for monitoring muscle oxidative metabolism in healthy subjects [19] and in hemiplegic patients [20] by using in house made devices. More recently, TD NIRS has been used for measurement of superficial and deep muscle deoxygenation during exercise [21,22,23] by using the TRS-20 device from Hamamatsu, but more application examples on muscle are still missing. 

In this paper, we present a novel TD NIRS instrument, designed for muscle applications in clinical environments, developed at our department. This novel TD NIRS medical device was thought for muscle oxidative metabolism monitoring. The main innovation introduced for this purpose is a custom 3D printed probe, able to host optical elements and 90° bended fiber optics for signal acquisition, developed for TD NIRS in vivo measurements. Although there are different examples of probes used in other TD NIRS applications, such as brain measurements, this is the first time that a TD NIRS system with a compact, integrated custom probe for in vivo muscle monitoring has been proposed. In the works already mentioned [19,20], prototypal probes were used, as they were made of rubber, hence not easily re-usable and not ideal to guarantee the necessary stability and repeatability of measurement performed. In this work, we addressed different problems such as skin adhesion, repeatability of acquisitions, reduction of motion artefacts, and disruption for the subject, by developing a new device that would be suitable for different exercise conditions and in a clinical setting. The system was widely characterized on solid phantoms and tested during in vivo applications. In particular, in Section 2, a brief description of TD NIRS data analysis is presented. In Section 3, the system and the custom probe for muscle measurements are described, and in Section 4, the system characterization is presented. In Section 5 we illustrate a series of in vivo experiments on healthy subjects to demonstrate the capability of this technique to perform real measurements on muscle. In particular, we will show a venous-arterial cuff occlusion, a cycling and an isometric. With these experiments, we show the capability of TD NIRS on muscle to provide measurements free from artifact movements, from surface physiological contaminations, and the sensitivity of the technique in monitoring different kind of hemodynamic variations in terms of type, amplitude, and execution rate.

## 2. TD NIRS Data Analysis

The typical in vivo NIRS measurements are implemented in the reflectance geometry, with the injection and collection fibers positioned on the same tissue’s surface. In the TD NIRS approach, we inject pulses of light into the tissue (with duration on the order of tens of picoseconds) and measure the photon distribution of time-of-flight (DTOF) after having travelled in the tissue itself. In order to estimate the optical properties, i.e., absorption and reduced scattering coefficients, a non-linear fitting procedure based on the Levenberg-Marquardt approach is implemented. The time-resolved reflectance curve is fitted with the analytical solution of the diffusion equation in a semi-infinite homogeneous geometry [24], previously convoluted with the instrument response function (IRF). From the absorption coefficients measured at two wavelengths in the 650–850 nm range and from the knowledge of the extinction coefficients of O_2_Hb and HHb [25], it is then possible to estimate the O_2_Hb and HHb concentrations in the tissue by exploiting the Lambert Beer’s law. Further, it is also possible to calculate the total hemoglobin content and the tissue oxygen saturation. During the data analysis process, we can discriminate the contribution coming from upper layers (UP) of the tissue from the one coming from deeper layers (DW) by using the information on photon path lengths [26,27]. This enables to distinguish between the O_2_Hb and HHb concentrations for the upper layers, which in the case of measurements on muscle is composed principally of skin, capillary bed, and fat layer, and deeper layers that contain the muscular fibers only. In order to apply this correction method, the a-priori knowledge of the thickness of the UP layer is necessary; it can be measured by means of a skin folder caliper or more accurately with an ecographic inspection.

## 3. TD NIRS Instrumentation

**Figure 1:**
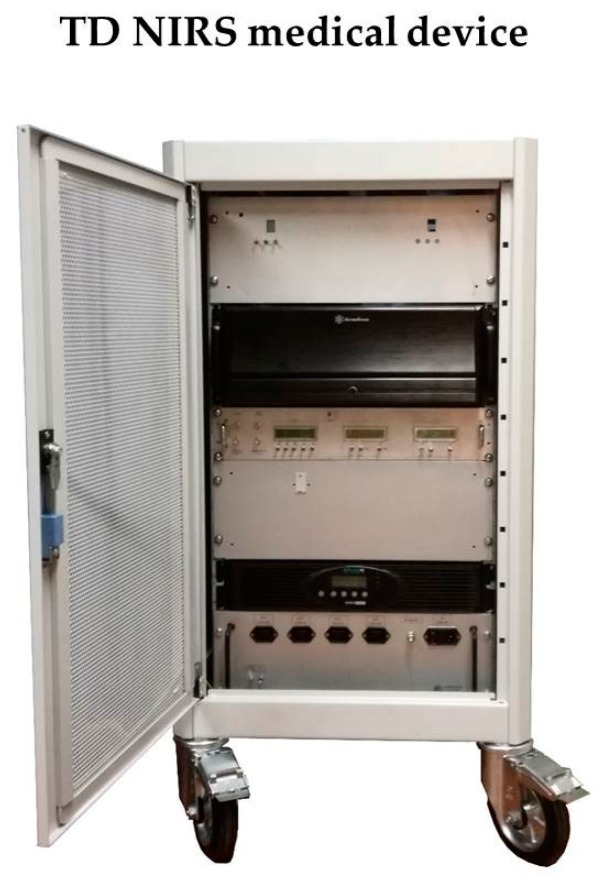
The time domain near infrared spectroscopy (TD NIRS) medical device.

The TD NIRS instrument for muscle oxidative metabolism monitoring is equipped with two pulsed diode lasers (LDH-P, PicoQuant GmbH, Berlin, Germany), electronically driven at 80 MHz (SEPIA II, PicoQuant), operating at 688 nm and 828 nm. Light is delivered into the tissue according to the wavelength Space Multiplexing (SM) principle [28]: laser pulses at the two different wavelengths are alternatively injected into the same location by means of a 2 × 2 optical switch (mol 2 × 2, LEONI Fiber Optics GmbH, Berlin, Germany) and recollected by two independent detection lines. This allows avoiding wavelength cross talk, increasing the signal-to-noise ratio. The detectors employed are hybrid photomultiplier tubes (HPM-100-50, Becker & Hickl GmbH, Berlin, Germany), while the signal is processed by two Time Correlated Single Photon Counting (TCSPC) boards (SPC-130, Becker & Hickl GmbH). Both the injection and detection lines are provided with light attenuation stages electronically controlled and optical system for light coupling. For technical details, it is possible to refer to our previous paper published in 2013 [29], where a similar set-up for brain imaging is presented. The system is suitable for laboratory measurement and for clinical settings acquisition, since it was structured as a class IIa medical device. It is in fact arranged in a compact and robust 19” rack case (60 × 60 × 108 cm) with four wheels and an Uninterruptible Power Supply (UPS) unit (Xanto S 1000R, OnLine USV-Systeme AG, Gruenwald, Germany) that besides protecting against interruption of external power supply, it also allows easy transfer from room to room in a clinical setting. The modularity structure allows the fast replacement of the different parts in case of failure or upgrade directly in clinic. Finally, a de-coupling transformer (REOMED 1000BV, Reo Italia s.r.l., Rezzato, BS, Italy) isolates the medical device from the external main power network and protects the hospital room electrical system from possible instrument malfunctioning. The device is shown in Figure 1.

For the injection optical fibers, we have chosen graded index multimode glass optical fibers with 100/140 μm of core/cladding and a numerical aperture of 0.29 (Lightech s.r.l., Capriate S.Gervasio, BG, Italy). For the detection optical fibers, we employed glass optical fiber bundles (Loptek Glasfasertechnick GmbH, Berlin, Germany) with an inner diameter of 3 mm and a numerical aperture of 0.57. We then developed a custom probe for custom fibers according to the specific application: when measurement on muscle are performed, it is important to have a good adhesion of the fibers on the tissue. To this purpose, the distal ends of the optical bundles are 90° bended to allow for wrapping by elastic bandage, and a custom system with a glass prism (PAG-RAP-05B-650-9-50, Lambda Research Optics Inc., Costa Mesa, CA, USA) is used coupled to the injection fibers in order to bend the injected light. The injection and collection optical fibers are hosted into two 3D printed supports created with a filament printer (Sharebot NG, Sharebot s.r.l., Nibionno, Italy) and a black PLA filament (3Ditaly, Roma, Italy). In this way, we obtained a stable mounting for one injection and two detection channels in each probe at 1.5 and 3.0 cm of interfiber distance ρ. 

**Figure 2:**
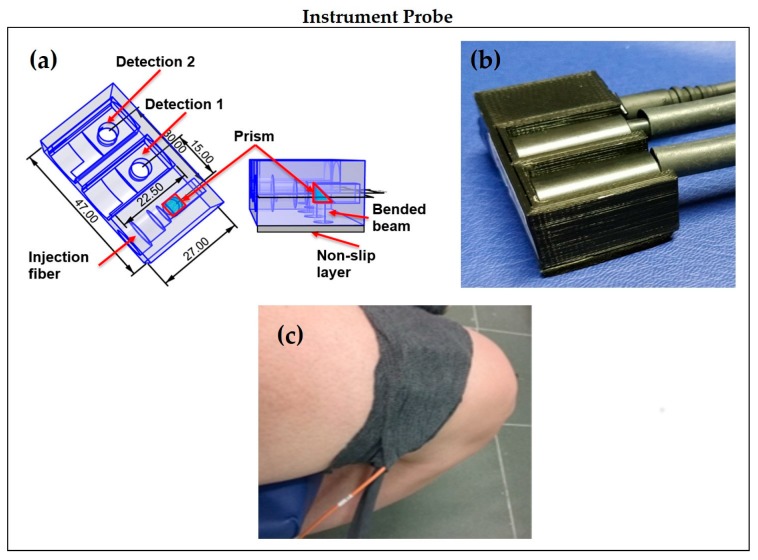
(**a**) Fiber probe schematic, with one injection and two detection optical fibers; (**b**) The 3D printed probe for measure with 90° bended optical fibers; (**c**) Black bandage for measurements on muscle.

In Figure 2a, a schematic of the probe is presented, while in Figure 2b, we show the 3D printed probe. Under the probe, a black biocompatible rubber is attached in order to improve the comfort when in contact with the patient’s skin and to screen the fiber inlet from the ambient light. It is eventually possible to add a biocompatible adhesive layer to firmly attach the probe to the muscle. A custom support was also designed and printed to allow the acquisition of the IRF, with injection and detection fibers directly faced. In addition, we complete the probe with a black elastic bandage that guarantees good adhesion and avoids the ambient light to reach the fibers, as demonstrated in the next sections, as shown in Figure 2c. The laser spot on the sample has a power density < 2 mW/mm^2^ according to the safety threshold [30].

## 4. Instrument Characterization 

In this section, the instrument characterization based on standardized protocols for TD NIRS is presented.

### 4.1. Basic IRF Characterization

**Figure 3:**
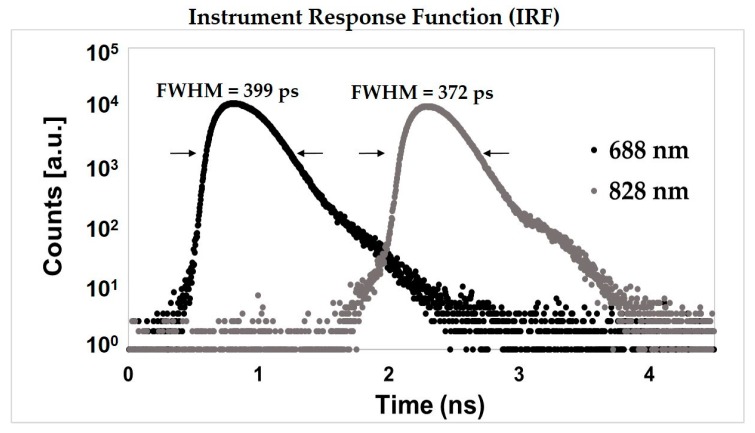
Instrument response function (IRF) for the red (688 nm) and infrared (828 nm) wavelength.

We explored, at first, the IRF characteristics. In Figure 3, an example of an IRF is presented. The Full Width at Half Maximum (FWHM) is about 400 ps and 370 ps at 688 nm and 828 nm, respectively. The two reflectance curves are typically acquired in two different temporal windows as usual in the SM modality. To obtain a stable IRF in terms of counts, peak position, and FWHM, the system needs a warm up-time of about 60 min (15 min) in order to have a stability in the range of the ±1% (±3%) of the final average value calculated over of the last 30 min of a 6-h long stability measurement in a room with an ambient temperature of 16 °C. 

### 4.2. Linearity on Solid Homogeneous Phantoms 

**Figure 4:**
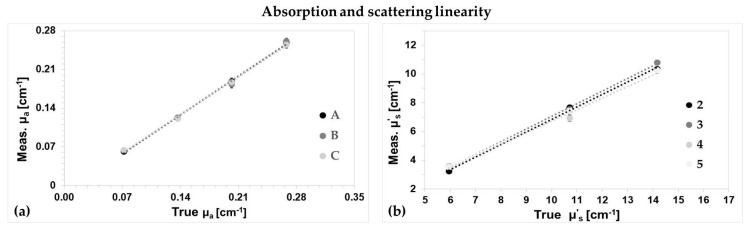
Measured absorption (**a**) and reduced scattering (**b**) coefficient as function of the true values for a series of calibrated homogeneous phantoms. Points are the average of five repeated measurements at 688 nm, and the error bars the standard deviations. Dotted lines represent the linear fit of the data series.

In this section, we investigate the capability of our system to retrieve the optical properties of a solid homogeneous phantom with known optical properties, as described in the MEDPHOT standardization protocol [31]. The phantoms are labeled with letters (A, B, C) and numbers (from 2 to 5) that represent, respectively, the reduced scattering (5, 10, 15 cm^−1^) and the absorption coefficient (from 0.07 to 0.28 cm^−1^, in 0.07 cm^−1^ step) values at a reference wavelength of 660 nm. We used a reflectance geometry, with one detection and one injection channel at ρ = 1.5 cm. In Figure 4 the linearity results at 688 nm are shown for the absorption and reduced scattering coefficients. In the x-axis the true values are represented, as obtained from a previous phantom characterization with a TD NIRS instrument [32] with a narrow (<100 ps) and stable IRF, which guarantee a good estimation of the optical parameters. In both plots each point represents the average values over five repeated measurements, with an acquisition time of 1 s, while the standard deviations are reported through the error bars and are always negligible with respect to the average values. Dotted lines are the linear fit over the points. Since the R^2^ is always larger than 0.98 for both μ_a_ and μ’_s_, we can affirm that the system shows an excellent linearity. Similar results were found for the other wavelength, 828 nm.

### 4.3. Deep Absorption Changes Localization 

As already discussed, muscular oxidative metabolism can be investigated by monitoring the oxy- and deoxy-hemoglobin concentration changes, which correspond from an optical point of view to the investigation of localized absorption changes at the selected wavelengths. In particular, it was demonstrated that black inclusions of different sizes in a homogeneous phantom could represent absorption perturbations of different entity [33]. For this purpose, we performed test on a homogeneous host phantom with a hole where it is possible to insert a movable cylinder with different PVC cylindrical black inclusions (3, 4, 5, and 7 mm of diameter) [34]. In particular, we assessed the instrument “sensitivity”, considering the measured contrast as a function of depth (Z-scan, measuring surface perpendicular to the inclusion movement) and the instrument “spatial resolution”, considering a lateral scan of the inclusion (Y-scan, measuring surface parallel to the inclusion movement) according to nEUROPT protocol [35]. The contrast is defined as

(1) $$C\left(t_{w},x_{inc}\right)\;=\;-ln\left[\frac{N\left(t_{w},x_{inc}\right)}{N_{0}(t_{w})}\right],$$

where *N*(*t_w_*,*x_inc_*) are the detected photons in a time window at the delay *t_w_*, with respect to the IRF peak position, while *N*_0_(*t_w_*) represents the number of photons in the same time window but for the inclusion in a reference position, typically far away from the measurement fibers. We calculated the contrast at 10 time window delays *t_w_*, 400 ps each, at 688 nm. The interfiber distance used was ρ = 3.0 cm, with 1 s acquisition time, and the scan was performed in 1 mm step. In general, we can assume that photons that travel only in the UP layer are detected at earlier time windows (early gates), while the ones that go more in depth in the DW layer are detected at later time windows (late gates).

**Figure 5:**
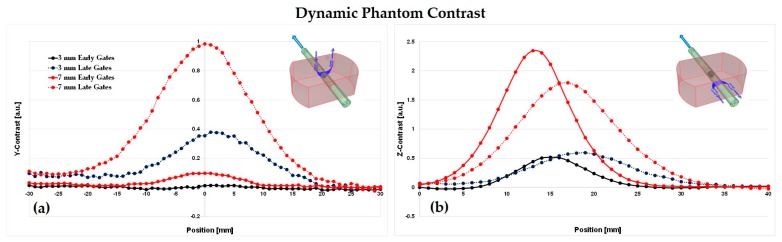
Contrast in function of the inclusion position. (**a**) Y-Scan: spatial resolution; (**b**) Z-Scan: depth resolution.

In Figure 5a,b, the results for the Y- and Z-Scan are shown, respectively. In both cases, we show the contrast for the biggest inhomogeneity (diameter = 7 mm, volume = 269 mm^3^ and equivalent Δμ_a_ = 0.40 cm^−1^) since in muscle oxidative metabolism we could find relatively large hemoglobin concentration (i.e., absorption) variations, such as during ischemia, and the smallest inhomogeneity (diameter = 3 mm, volume 21 mm^3^ and equivalent Δμ_a_ = 0.05 cm^−1^) in order to test the lowest sensitivity limit of the instrument. With solid lines, we represent the curves for early time window (Y-scan: time windows from 400 to 1200 ps; Z-Scan: time windows from 400 to 1200 ps for the smallest inclusion and time windows from 0 to 800 ps for the biggest one), instead with dotted lines the late time window (Y-Scan: time windows from 2000 to 2800 ps; Z-Scan: time windows from 1600 to 2400 ps for the smallest inclusion and time windows from 2400 to 3200 ps for the biggest one). In both the scans, the contrast is higher for the large inclusion, as expected. For the Y-Scan at the zero-point position, when the inclusion is exactly under the source-detector pair’s plan, the contrast has a maximum, in particular for the late time window, which corresponds to those photons that traveled deeper in the phantom. For the early time windows, the contrast is smaller, being almost equal to zero for the smallest inclusion: these photons only travel close to the phantom’s surface and cannot reach the inclusion. Moving the inclusion far from the optical fibers, the contrast decreases until it reaches values around zero (at about ±30 mm far away from the central position). 

For the Z-Scan, we should consider the zero-position where the inclusion is on the phantom’s border coincident with the entrance surface. In this case, the highest contrast is reached at different inclusion positions for early and late time windows. In particular, for early time windows, the contrast peak occurs at lower depths, showing an excellent depth selectivity. 

### 4.4. Reproducbility and Repeatibility on Solid Homogeneous Phantom

Accordingly to the MEDPHOT protocol [31] to assess the instrument reproducibility, we performed ten repeated 1 s measurements, for three following days, on a solid homogeneous phantom with known optical properties (μ_a_ = 0.1 cm^−^^1^ and μ’_s_ = 10 cm^−^^1^ at 660 nm). The percentage Coefficient of Variation (CV) obtained for the absorption and reduced scattering was always <0.9% and <1.6%, respectively, for all the wavelengths and interfiber distances. It was calculated considering the average through the days. 

For the in vivo applications, it is also important to assess the repeatability of the instrument measurement when the probe is repetitively re-positioned on the same point. For this purpose, we performed five trials of 10 repeated measurements on the same phantom described above and with the same geometry. Between each trial, we remove and then re-collocated the probe on the phantom. We calculated the CV for the absorption and reduced scattering coefficient obtaining always a CV<1.2%, for all the wavelengths and interfiber distances. 

## 5. In Vivo Tests

In this section, we will show some representative in vivo applications on the human muscular tissue in order to show all the potentiality of this technique. All subjects gave their informed consent for inclusion before they participated in the tests. The study was conducted in accordance with the Declaration of Helsinki, and the protocol was approved by the Ethics Committee of Politecnico di Milano (Project identification code: fNIRS 2016). At first, we tested the in vivo repeatability of the measurements (Section 5.1) to quantify the error that we can have repositioning the probe on a living subject. Then we performed a cuff occlusion of an arm to show the ability of the system to follow the tissue hemodynamics changes (Section 5.2). In Section 5.3 and Section 5.4, we report on a cycling and an isometric exercise, respectively. During these two experiments, we can observe the ability to detect hemodynamics parameters without being affected by movement artifacts and the sensibility of the technique to detect changes with different kind of task modulations. We always used one injection-one detection scheme, with an interfiber distance of 1.5 cm. 

### 5.1. In Vivo Repeatability 

We performed six trials of 30 repeated measurements on the gastrocnemius muscle of one subject. Between each trial, we remove and then re-collocated the probe on the same point. The percentage CV for the absorption and reduced scattering coefficient were <3% for both the wavelengths. As expected, the CV values measured on living subjects are higher than the ones found for phantoms (see Section 4.4) due to the physiological variability. 

### 5.2. Venous-Arterial Arm Occlusion

**Figure 6:**
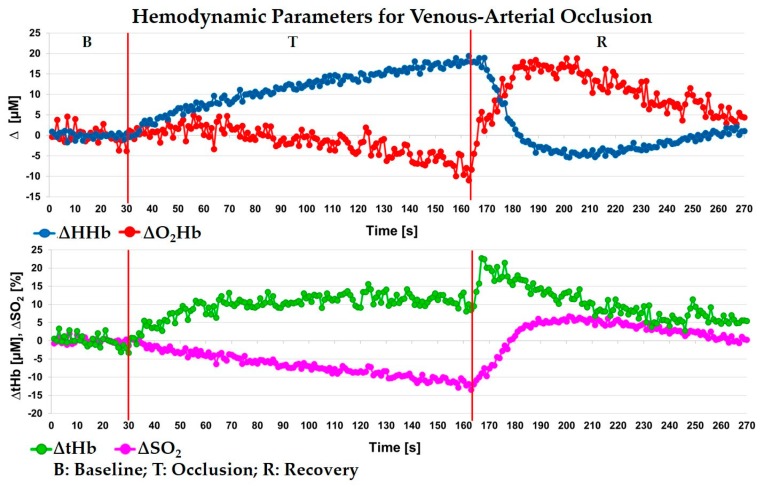
Venous-arterial arm occlusion. Variations of the hemodynamic parameters respect to the baseline for the down (DW) layer. O_2_Hb: oxy-hemoglobin, HHb: deoxy-hemoglobin, tHb: total hemoglobin and SO_2_: oxygen saturation.

A venous-arterial cuff occlusion of the arm muscle was performed on a healthy subject (male, 39 years old). The probe was positioned on the brachial muscle of the left arm. The protocol consisted of 30 s baseline (B), 135 s occlusion (increasing pressure, max pressure equal to 300 mmHg) (T) and 105 s recovery (R) after the cuff release. Each wavelength was acquired for 0.5 s determining a sampling rate of 1 s. With a skin folder caliper, we measured 5 mm of thickness for the UP layer under the probe. The arm hemodynamic parameters averaged over the 30 s of baseline were: O_2_Hb = 86 ± 2 μM, HHb = 32 ± 1 μM, tHb = 119 ± 2 μM, and SO_2_ = 73 ± 1%. The changes of these parameters with respect to the baseline during the whole acquisition are shown in Figure 6 for the DW layer. A moving average filter of the 3rd order was applied. Since the cuff was inflated quite slowly, at the beginning the pressure applied was only enough to close veins and not arteries, meaning that oxygenated blood could still enter into the occluded part, while there was not wash out of de-oxygenated blood (increasing of HHb and a quite stable value for O_2_Hb).

After 40 s, higher pressure values of the cuff were reached and arteries were closed (ischemia) with a consequent decrease in O_2_Hb concentration. The tHb has an initial increase related to vein occlusion, but once the arterial compartment is also occluded, a constant blood volume is observed, as expected. The SO_2_ well reflects the tissue oxygenation state, since it has a decreasing trend during the whole T period. After the cuff release, we can observe the typical hyperemia peak for all the hemodynamic parameters, then they slowly go back to the initial condition. However, the recovery time was not enough to return to the baseline values.

### 5.3. Cycling Exercise on Vastus Lateralis

**Figure 7:**
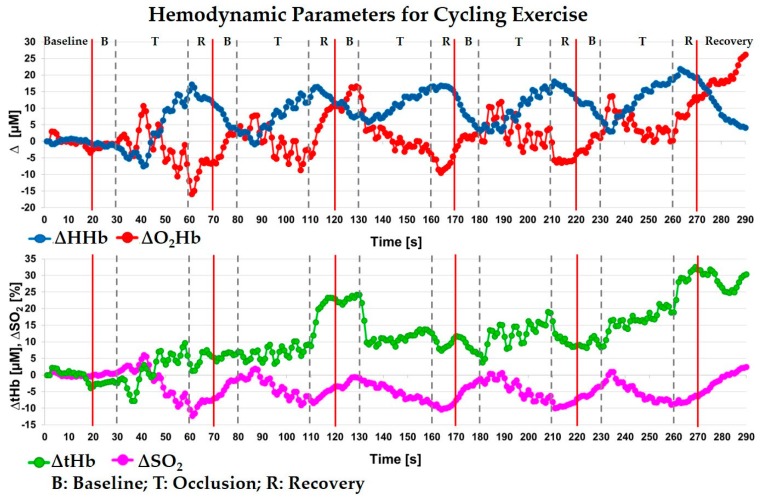
Variations, with respect to the baseline, of the hemodynamic parameters measured on the vastus lateralis muscle during the cycling exercise, for the DW layer. O_2_Hb: oxy-hemoglobin, HHb: deoxy-hemoglobin, tHb: total hemoglobin and SO_2_: oxygen saturation.

In this section, we show the results obtained during a cycling exercise with the probe placed on the right vastus lateralis muscle of a healthy volunteer (male, 22 years old). Each wavelength was acquired for 0.5 s determining a sample rate of 1 s. The experiment consisted on an initial baseline period of 20 s, during which the subject was not moving. Then, for five times, the subject was asked to perform the following trial: 10 s of baseline (B) without movement, 30 s of cycling (T) with a pedaling frequency of about 70 rpm, and 10 s of recovery (R) again without movement. A final recovery period of 20 s ends the experiment. The whole experiment length was 290 s. The custom probe was firmly positioned over the muscle, and we observed no movement or detachment during the exercise. The baseline values of the hemodynamic parameters were: O_2_Hb = 99 ± 2 μM, HHb = 37 ± 1 μM, tHb = 136 ± 2 μM and SO_2_ = 73 ± 1%. In Figure 7, the hemodynamic variations with respect to the initial 20 s baseline for the only DW layer are shown during the whole exercise. A moving average filter of the 3rd order was applied. During each cycling period (T), the HHb and O_2_Hb are initially quite flat and after about 8 s they are respectively increasing and decreasing. This behavior is similar to the one shown by Grassi et al. [36] during the transition from an unloaded pedaling to a constant-load exercise. During the following recovering periods (R), we observe the opposite behavior. As expected, SO_2_ during the task is decreasing. The blood volume (green line, tHb) shows an increasing trend if we consider the total experiment length, denoting probably a vasodilation in the muscular region.

### 5.4. Isometric Exercise on Vastus Lateralis

In this experiment, we present an application on the right vastus lateralis muscle of an adult volunteer (female, 33 years old) who sat on a chair during an isometric exercise. The acquisition time was 1 s (0.5 s for each wavelength). The measured thickness of the UP layer was 7 mm. The experiment presents an initial 30 s of baseline, then the subject had to perform rhythmically isometric knee extensions at maximum voluntary contraction (MVC) against a fixed orthopedic elastic band kept fixed to her ankle. The subject maintained the same leg position and angle inclination during the whole exercise. The measured baseline hemodynamic parameters were: O_2_Hb = 33 ± 1 μM, HHb = 13 ± 1 μM, tHb = 46 ± 1 μM and SO_2_ = 72 ± 1%.

**Figure 8:**
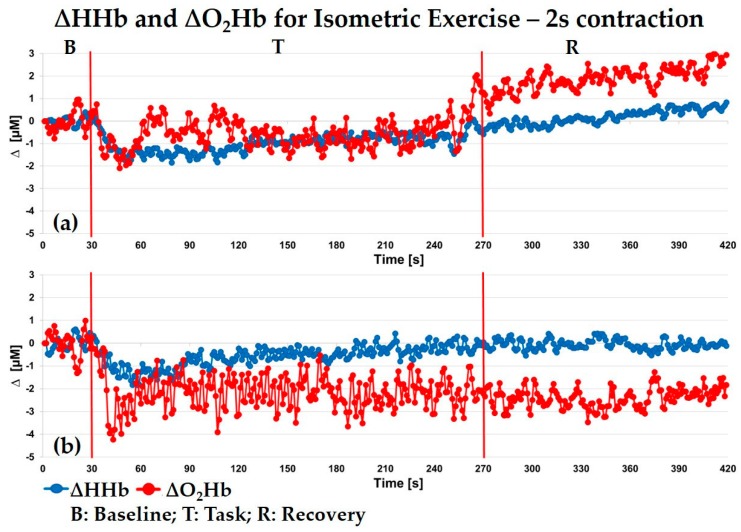
Isometric exercise with 2 s repetition rate on vastus lateralis muscle. Variations of oxy- (O_2_Hb) and deoxy-hemoglobin (HHb) respect to the baseline for the UP (**a**) and DW layer (**b**). B = baseline, T = task and R = recovery.

During the experiment, contractions for 240 s with a repetition rate of 2 s were performed, followed by 150 s of recovery. In Figure 8, the variations with respect to the initial baseline values for O_2_Hb and HHb for the UP (a) and DW (b) layer are shown, respectively. A moving average filter of the 3rd order was applied. Since the thickness of the UP layer is 7 mm and the hemodynamic variations are modest, we can state that the error committed in the estimation of the parameters for both UP and DW layers is below 3% [27]. Since the sampling time is 1 s and the contraction is 2 s long, we cannot follow the hemodynamic changes due to each single muscular contraction and relaxation but rather the whole trend during the exercise period (T), allowing the assessment of the hemodynamics kinetics during the whole experiment, which is a relevant parameters to be assessed in physiological evaluation [37]. In the DW layer, Figure 8b, we can observe an initial rapid decrease in O_2_Hb, followed by an increase; after about 30 s from the starting of contractions O_2_Hb reaches a plateue (ΔO_2_Hb = 2 μM). Then, its value never returned to the baseline, also during the recovery period (R). The HHb has a much slower initial decrease until 30 s after the task onset, then an increase takes place, reaching the baseline value about after 100 s. This trend is in agreement with previous studies by CW NIRS [38]. In the UP layer, Figure 8a, we can observe a decrease of O_2_Hb in the first 30 s after the beginning of the task (maximum variation about 2 μM). This is followed by an overshoot of O_2_Hb. Then, its concentration keeps oscillating around the baseline values. After 60 s, the mean value is reduced again by 1 μM until the last seconds of the task, when it starts to increase againg. For what concern HHb, we highlight a decrease of about 2 μM in the first 30 s, followed by a slow increasing trend towards initial values. Finally, during the whole recovery phase we observe that both O_2_Hb and HHb keep increasing (with a different slope), beacause of all these behaviours, the kinetics appears to reflect the perfusion of skin and superficial layer capillary bed in response to the exercise rather than the muscular oxidative metabolism.

## 6. Conclusions

In this paper, we have presented for the first time a TD NIRS medical device tailored for muscular oxidative state monitoring. The instrument has a custom 3D-printed probe that facilitates its use on moving legs and arms. The instrument was characterized in terms of linearity in the estimate of the optical properties of calibrated phantoms and of reproducibility (on phantoms and in vivo). We also tested the spatial and depth selectivity on a dynamic phantom. The device was employed during different in vivo measurements in order to assess its capability to acquire stable and free of movement artifact signal and to estimate the hemodynamic variations in particular during cuff occlusion, cycling and isometric exercises. As already presented by the same authors in 2016 [39], a new generation of detectors could be an improvement of this medical device. In Ref 39 the possibility of employing fiber-free SiPM detectors for in vivo TD fNIRS acquisitions is shown. These detectors can be put directly in contact with the patient’s skin, avoiding the use of optical fibers, which broaden the temporal response of the TD system, have a reduced collection capability and attenuate the collected signal.
